# First-line nivolumab plus ipilimumab combined with two cycles of chemotherapy in advanced non-small cell lung cancer: a subanalysis of Asian patients in CheckMate 9LA

**DOI:** 10.1007/s10147-022-02120-0

**Published:** 2022-02-19

**Authors:** Thomas John, Hiroshi Sakai, Satoshi Ikeda, Ying Cheng, Kazuo Kasahara, Yuki Sato, Yoshiro Nakahara, Masayuki Takeda, Hiroyasu Kaneda, Helong Zhang, Makoto Maemondo, Koichi Minato, Takeshi Hisada, Yuki Misumi, Miyako Satouchi, Katsuyuki Hotta, Ang Li, Abderrahim Oukessou, Shun Lu

**Affiliations:** 1 Austin Hospital, 145 Studley Road, Heidelberg, VIC 3084 Australia; 2 Saitama Cancer Center, 780, Komuro, Ina, Kitaadachi District, Saitama, 362-0806 Japan; 3 Kanagawa Cardiovascular and Respiratory Center, 6 Chome-16-1 Tomiokahigashi, Kanazawa Ward, Yokohama, Kanagawa 236-0051 Japan; 4 Jilin Cancer Hospital, No. 1018 Huguang Road, Changchun, 130012 China; 5 Kanazawa University Hospital, 13-1 Takaramachi, Kanazawa, Ishikawa 920-8641 Japan; 6 Kobe City Medical Center General Hospital, 2-1-1, Minatojima Minamimachi, Chuo Ward, Kobe, Hyogo 650-0047 Japan; 7 Kanagawa Cancer Center, 2 Chome-3-2 Nakao, Asahi Ward, Yokohama, Kanagawa 241-8515 Japan; 8 Kindai University Hospital, 377-2 Onohigashi, Osakasayama, Osaka 589-8511 Japan; 9 Osaka City University Hospital, 1 Chome-5-7 Asahimachi, Abeno Ward, Osaka, 545-8586 Japan; 10 Tangdu Hospital, Xinsi Road, Baqiao District, Xi’an, 710038 Shaanxi China; 11 Iwate Medical University Hospital, 2-1-1 Idaidori, Yahaba-cho, Shiwa District, Iwate, 028-3695 Japan; 12 Gunma Prefectural Cancer Center, 617-1 Takahayashinishi-cho, Ota-shi, Gunma, 373-8550 Japan; 13 Gunma University Hospital, 3 Chome-39-15 Showamachi, Maebashi, Gunma 371-8511 Japan; 14 Yokohama Municipal Citizen’s Hospital, 1-1 Mitsuzawa Nishimachi, Kanagawa Ward, Yokohama, Kanagawa 221-0855 Japan; 15 Hyogo Cancer Center, 13-70 Kita-Oji, Akashi, Hyogo 673-8558 Japan; 16 Okayama University Hospital, 2 Chome-5-1 Shikatacho, Kita Ward, Okayama, 700-0914 Japan; 17 Bristol Myers Squibb, 3551 Lawrenceville Road, Princeton, NJ USA; 18 Shanghai Lung Cancer Center, Shanghai Chest Hospital, Shanghai Jiao Tong University, 241 West Huaihai Road, Shanghai, China

**Keywords:** Immunotherapy, Non-small cell lung cancer, Asia, Japan, Nivolumab, Ipilimumab

## Abstract

**Background:**

CheckMate 9LA, a phase 3, randomized, open-label study in first-line advanced non-small cell lung cancer (NSCLC), showed significantly improved overall survival (OS) with nivolumab plus ipilimumab combined with 2 cycles of chemotherapy versus chemotherapy alone (4 cycles). We present results for the Asian subpopulation enrolled in Japan and China.

**Methods:**

Patients aged ≥ 18 years with treatment-naive, histologically confirmed stage IV or recurrent NSCLC, Eastern Cooperative Oncology Group performance status 0–1 and no sensitizing *EGFR/ALK* mutations were randomized 1:1 to nivolumab [360 mg every 3 weeks (Q3W)] plus ipilimumab (1 mg/kg Q6W) combined with chemotherapy (Q3W for 2 cycles), or chemotherapy alone (Q3W for 4 cycles). Primary endpoint was OS; secondary endpoints included progression-free survival (PFS) and objective response rate (ORR).

**Results:**

Twenty-eight patients received nivolumab plus ipilimumab combined with chemotherapy and 30 received chemotherapy. At a minimum follow-up of 12.7 months, median OS was not reached with nivolumab plus ipilimumab combined with chemotherapy versus 13.3 months with chemotherapy [hazard ratio (HR) 0.33; 95% confidence interval (CI) 0.14–0.80]. Median PFS was 8.4 versus 5.4 months (HR 0.47; 95% CI 0.24–0.92) and ORR was 57% versus 23%, respectively. Grade 3–4 treatment-related adverse events were observed in 57% versus 60% of patients, respectively.

**Conclusion:**

Consistent with results in the all randomized population, nivolumab plus ipilimumab combined with chemotherapy improved efficacy in the Asian subpopulation versus chemotherapy alone and had a manageable safety profile, supporting its use as first-line treatment for advanced NSCLC in Asian patients.

**Supplementary Information:**

The online version contains supplementary material available at 10.1007/s10147-022-02120-0.

## Introduction

Clinical trials of immunotherapy regimens have demonstrated improvements in clinical outcomes over traditional chemotherapy for patients with advanced non-small cell lung cancer (NSCLC) [1–5]. Immunotherapy treatments are now approved as monotherapy or in combination with other immunotherapy agents or chemotherapy for the first-line treatment of advanced NSCLC in many countries [6–8]. The immunotherapy agents nivolumab and ipilimumab have distinct but complementary mechanisms of action: nivolumab, a fully human immunoglobulin G4 programmed death (PD)-1 immune checkpoint inhibitor antibody, restores anti-tumor T-cell function [9–11] and ipilimumab, a fully human immunoglobulin G1 cytotoxic T-lymphocyte antigen-4 immune checkpoint inhibitor antibody, induces T-cell proliferation and de novo anti-tumor T-cell responses, including an increase in memory T cells [12–14].

First-line treatment with nivolumab plus ipilimumab provides durable long-term survival benefit for patients with advanced NSCLC, regardless of tumor PD-L1 expression and histology, as observed in CheckMate 227 [1, 15]. Addition of a limited course (2 cycles) of platinum-doublet chemotherapy to nivolumab plus ipilimumab was hypothesized to provide rapid initial disease control while potentially building on the durable benefits of nivolumab plus ipilimumab. CheckMate 9LA (NCT03215706), a phase 3, randomized, open-label study in first-line advanced NSCLC, showed significantly improved overall survival (OS), progression-free survival (PFS), and objective response rate (ORR) with nivolumab plus ipilimumab combined with 2 cycles of chemotherapy versus 4 cycles of chemotherapy alone, with a manageable safety profile [16, 17]. These data led to the approval of nivolumab plus ipilimumab combined with chemotherapy (2 cycles) in many countries, including the United States, the European Union, and several Asian countries such as Japan, South Korea, Taiwan, and Singapore, as first-line treatment for patients with metastatic or recurrent NSCLC, with no *EGFR* or *ALK* genomic tumor aberrations [7, 8, 18–22].

Differences in the treatment outcomes and safety profiles of various therapies have been observed between Asian and non-Asian patients with NSCLC [23–25]. It is therefore important to evaluate the clinical activities of therapies in Asian populations to optimize treatment strategies. Here, we report efficacy and safety data for the Asian subpopulation in CheckMate 9LA.

## Patients and methods

### Patients and treatment

The study design for CheckMate 9LA (NCT03215706) has been described previously [16]. Briefly, eligible patients were aged ≥ 18 years with treatment-naive, histologically confirmed stage IV or recurrent NSCLC, an Eastern Cooperative Oncology Group (ECOG) performance status of 0–1, and no sensitizing *EGFR* or *ALK* mutations. Patients in this subanalysis were enrolled at 16 centers (14 centers in Japan and 2 in China).

Patients were randomly assigned (1:1) by an interactive web response system via permuted blocks (block size of 4) to either: nivolumab [360 mg intravenously every 3 weeks (Q3W)] plus ipilimumab (1 mg/kg intravenously Q6W) combined with histology-based, platinum-doublet chemotherapy (intravenously Q3W for 2 cycles), or histology-based chemotherapy alone (Q3W for 4 cycles). Randomization was stratified by tumor histology (squamous versus nonsquamous), sex, and tumor programmed death ligand-1 (PD-L1) expression (< 1% versus ≥ 1%). In either arm, patients with nonsquamous disease received pemetrexed plus cisplatin or pemetrexed plus carboplatin, and those with squamous disease received paclitaxel plus carboplatin. Patients with nonsquamous histology in the chemotherapy arm could receive optional pemetrexed maintenance until disease progression or unacceptable toxicity. For patients in the nivolumab plus ipilimumab combined with chemotherapy arm, immunotherapy treatment continued until disease progression, unacceptable toxicity, or completion per protocol (2 years).

### Endpoints and assessments

The primary endpoint of CheckMate 9LA was OS in all randomly assigned patients. Secondary endpoints included PFS by blinded independent central review (BICR) and ORR by BICR. Safety was analyzed in all treated patients, and adverse events (AEs) were graded per the National Cancer Institute Common Terminology Criteria for Adverse Events (version 4.0). Treatment-related adverse events (TRAEs) and treatment-related select AEs, defined as AEs with potential immunologic cause, were collected between first dose and 30 days after last dose of study drug. Tumor PD-L1 expression was determined by the PD-L1 IHC 28-8 pharmDx assay (Agilent Technologies, Inc., Santa Clara, CA, USA). All endpoints described above were assessed for the Asian subpopulation in this analysis.

### Statistical analyses

OS, PFS, and duration of response (DOR) were assessed by Kaplan–Meier method. Hazard ratios (HRs) for OS and PFS were calculated using a Cox proportional hazards model with treatment group as a single covariate. All subgroup analyses were descriptive; no formal statistical testing was done for the Asian subpopulation as these were post hoc analyses.

## Results

### Patients

**Fig. 1:**
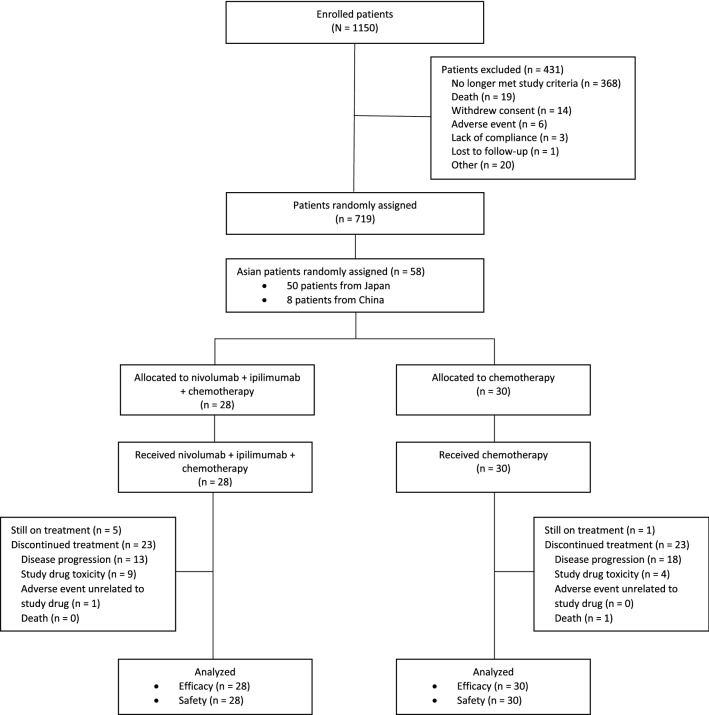
Consolidated Standards of Reporting Trials (CONSORT) diagram of patient disposition in the Asian subpopulation of CheckMate 9LA

In the all randomized study population of CheckMate 9LA, 361 patients were randomized to nivolumab plus ipilimumab combined with chemotherapy and 358 were randomized to chemotherapy. In the Asian subpopulation, 28 patients (22 patients from Japan and 6 from China) were randomized to nivolumab plus ipilimumab combined with chemotherapy and 30 patients (28 patients from Japan and 2 from China) to chemotherapy (Fig. 1). This analysis was based on a database lock of March 9, 2020, with minimum follow-up of 12.7 months for OS and 12.2 months for all other endpoints. Baseline characteristics in Asian patients were generally balanced between treatment groups (Table 1), although the number of patients was small in some subgroups. Median age was 68 years in the nivolumab plus ipilimumab combined with chemotherapy group, and 66 years in the chemotherapy group. Most patients in both the nivolumab plus ipilimumab combined with chemotherapy and chemotherapy treatment arms were current/former smokers (93% and 87%, respectively) and had nonsquamous histology (61% and 70%, respectively). All patients in the Asian subpopulation had quantifiable tumor PD-L1 expression; most had tumor PD-L1 expression ≥ 1% (57% in each arm).

**Table 1:** Baseline demographic and clinical characteristics of the Asian subpopulation in CheckMate 9LA

	NIVO + IPI + chemo^a^ (*n* = 28)	Chemo^b^ (*n* = 30)
Age, median (range), years	68 (46–77)	66 (31–77)
< 65 years, *n* (%)	10 (36)	11 (37)
≥ 65 to < 75 years, *n* (%)	16 (57)	16 (53)
≥ 75 years, *n* (%)	2 (7)	3 (10)
Female, *n* (%)	3 (11)	6 (20)
Region, *n* (%)		
Japan	22 (79)	28 (93)
China	6 (21)	2 (7)
Disease stage, *n* (%)		
Stage IV	24 (86)	27 (90)
Recurrent	4 (14)	3 (10)
ECOG performance status, *n* (%)		
0	10 (36)	12 (40)
1	18 (64)	18 (60)
Smoking status, *n* (%)		
Never smoker	2 (7)	4 (13)
Current/former smoker	26 (93)	26 (87)
Histology, *n* (%)		
Squamous	11 (39)	9 (30)
Nonsquamous	17 (61)	21 (70)
Bone metastasis, *n* (%)	9 (32)	10 (33)
CNS metastasis, *n* (%)	6 (21)	5 (17)
Liver metastasis, *n* (%)	2 (7)	5 (17)
Tumor PD-L1 expression,^c^ *n* (%)		
< 1%	12 (43)	13 (43)
≥ 1%	16 (57)	17 (57)
1–49%	9 (32)	9 (30)
≥ 50%	7 (25)	8 (27)

*Chemo* chemotherapy, *CNS* central nervous system, *ECOG* Eastern Cooperative Oncology Group, *IPI* ipilimumab, *n* number of patients, *NIVO* nivolumab, *PD-L1* programmed death ligand-1

^a^Nivolumab plus ipilimumab combined with chemotherapy (2 cycles)

^b^Chemotherapy alone (4 cycles, with optional pemetrexed maintenance for nonsquamous histology)

^c^Quantifiable for 100% of Asian patients

### Treatment exposure and subsequent therapy

At the database lock, 18% of Asian patients were still receiving nivolumab plus ipilimumab combined with chemotherapy; 3% of patients were still receiving chemotherapy (Table 2). The most common reasons for treatment discontinuation (in ≥ 10% of patients) were disease progression (46% of patients in the nivolumab plus ipilimumab combined with chemotherapy arm and 60% of patients in the chemotherapy arm) and study drug toxicity (32% and 13%, respectively). In the nivolumab plus ipilimumab combined with chemotherapy arm, median duration of therapy was 4.2 months and 93% of patients completed 2 cycles of chemotherapy. In the chemotherapy arm, median duration of therapy was 2.1 months; 63% of patients received 4 or more cycles of treatment; 57% of patients with nonsquamous histology received pemetrexed maintenance. In patients with a PFS event (progression or death or censored for initiation of subsequent systemic therapy), subsequent systemic therapy was received by 77% of patients in the nivolumab plus ipilimumab combined with chemotherapy arm and 79% of patients in the chemotherapy arm; subsequent immunotherapy was received by 9% and 69% of patients, respectively; and subsequent chemotherapy was received by 73% and 38% of patients, respectively (Table 3). Subsequent therapies received among all patients are reported in Table 3.

**Table 2:** Treatment exposure in the Asian subpopulation of CheckMate 9LA

	NIVO + IPI + chemo^a^ (*n* = 28)	Chemo^b^ (*n* = 30)
Duration of therapy, median (range), months	4.2 (0.0–17.6)	2.1 (0.0–15.9)
Number of doses received, median (range)		
Nivolumab	7 (1–25)	NA
Ipilimumab	4 (1–13)	
Cycles of platinum-based chemotherapy received, *n* (%)		
1	2 (7)	1 (3)
2	26 (93)	5 (17)
3	0	5 (17)
≥ 4^c^	0	19 (63)
Patients receiving pemetrexed maintenance therapy,^d^ *n* (%)	NA	12^e^ (40)
Patients still on treatment, *n* (%)	5 (18)	1 (3)

^a^Nivolumab plus ipilimumab combined with chemotherapy (2 cycles)

^b^Chemotherapy alone (4 cycles, with optional pemetrexed maintenance for nonsquamous histology)

^c^Includes patients who completed 4 cycles of platinum-doublet chemotherapy and patients with nonsquamous histology who received pemetrexed maintenance

^d^Pemetrexed maintenance therapy was optional for patients with nonsquamous histology

^e^57% of patients with nonsquamous histology

*Chemo* chemotherapy, *IPI* ipilimumab, *n* number of patients, *NA* not available, *NIVO* nivolumab

**Table 3:** Subsequent therapy^a^ received in the Asian subpopulation of CheckMate 9LA

Subsequent therapy, *n* (%)	Asian patients		Asian patients with a PFS event per BICR^d^	
	NIVO + IPI + chemo^b^ (*n* = 28)	Chemo^c^ (*n* = 30)	NIVO + IPI + chemo^b^ (*n* = 22)	Chemo^c^ (*n* = 29)
Any subsequent therapy	18 (64)	25 (83)	18 (82)	25 (86)
Subsequent radiotherapy	6 (21)	11 (37)	6 (27)	11 (38)
Subsequent systemic therapy	17 (61)	23 (77)	17 (77)	23 (79)
Immunotherapy	2 (7)	20 (67)	2 (9)	20 (69)
Anti–PD-1	0	15 (50)	0	15 (52)
Anti–PD-L1	1 (4)	4 (13)	1 (4)	4 (14)
Other immunotherapy	1 (4)	2 (7)	1 (4)	2 (7)
VEGFR inhibitors	6 (21)	6 (20)	6 (27)	6 (21)
Other systemic therapy—experimental	1 (4)	1 (3)	1 (4)	1 (3)
Other systemic therapy—chemotherapy	16 (57)	11 (37)	16 (73)	11 (38)

*BICR* blinded independent central review, *Chemo* chemotherapy, *IPI* ipilimumab, *n* number of patients, *NIVO* nivolumab, *PD-1* programmed death-1, *PD-L1* programmed death ligand-1, *PFS* progression-free survival, *VEGFR* vascular endothelial growth factor receptor

^a^Defined as therapy initiated on or after first dosing date (or randomization date if the patient was never treated); patients may have received more than 1 type of subsequent therapy

^b^Nivolumab plus ipilimumab combined with chemotherapy (2 cycles)

^c^Chemotherapy alone (4 cycles, with optional pemetrexed maintenance for nonsquamous histology)

^d^Includes patients that had an event of progression or death as well as being censored for subsequent systemic therapy

### Efficacy outcomes

**Fig. 2:**
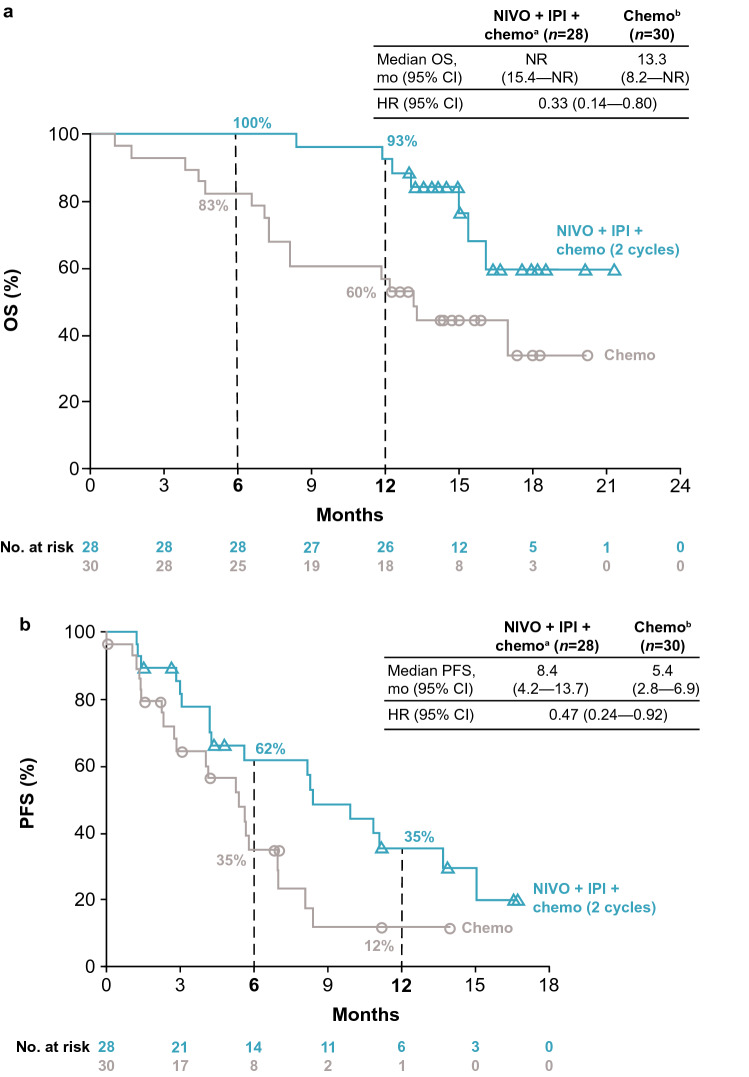
OS (**a**) and PFS per BICR (**b**) in the Asian subpopulation of CheckMate 9LA. ^a^Nivolumab plus ipilimumab combined with chemotherapy (2 cycles); ^b^Chemotherapy alone (4 cycles, with optional pemetrexed maintenance for nonsquamous histology). *BICR* blinded independent central review, *chemo* chemotherapy, *CI* confidence interval, *HR* hazard ratio, *IPI* ipilimumab, *mo* months, *n* number of patients, *NIVO* nivolumab, *NR* not reached, *OS* overall survival, *PFS* progression-free survival

OS was improved with nivolumab plus ipilimumab combined with chemotherapy versus chemotherapy alone among Asian patients (Fig. 2a). Median OS was not reached [95% confidence interval (CI) 15.4–not reached] for patients in the nivolumab plus ipilimumab combined with chemotherapy arm versus 13.3 months (8.2–not reached) for patients in the chemotherapy arm; HR 0.33 (95% CI 0.14–0.80); 6-month and 1-year OS rates were 100% (95% CI 100.0–100.0) and 93% (95% CI 74.3–98.2) with nivolumab plus ipilimumab combined with chemotherapy versus 83% (95% CI 64.5–92.7) and 60% (95% CI 40.5–75.0) with chemotherapy, respectively. OS by histology (squamous and nonsquamous) and tumor PD-L1 expression (< 1% and ≥ 1%) also favored nivolumab plus ipilimumab combined with chemotherapy compared with chemotherapy (Online Resource 1).

PFS per BICR in Asian patients was improved with nivolumab plus ipilimumab combined with chemotherapy versus chemotherapy alone**.** Median PFS was 8.4 months (95% CI 4.2–13.7) for patients in the nivolumab plus ipilimumab combined with chemotherapy arm versus 5.4 months (95% CI 2.8–6.9) for patients in the chemotherapy arm; HR 0.47; 95% CI 0.24–0.92 (Fig. 2b); 6-month and 1-year PFS rates were 62% (95% CI 40.2–77.3) and 35% (95% CI 17.1–53.9) with nivolumab plus ipilimumab combined with chemotherapy versus 35% (95% CI 17.0–53.0) and 12% (95% CI 2.1–29.9) with chemotherapy, respectively.

ORR and DOR per BICR are presented in Table 4. ORR was higher with nivolumab plus ipilimumab combined with chemotherapy (16 of 28 patients, 57%) versus chemotherapy alone (7 of 30 patients, 23%). Two patients (7%) achieved complete response with nivolumab plus ipilimumab combined with chemotherapy; no complete responses were reported in the chemotherapy arm. Median time to response for patients who received nivolumab plus ipilimumab combined with chemotherapy was 1.5 months versus 2.8 months for those who received chemotherapy. Median DOR was longer with nivolumab plus ipilimumab combined with chemotherapy [7.0 months (95% CI 2.8–11.0)] than with chemotherapy [4.4 months (95% CI 2.8–not reached)].

**Table 4:** ORR and DOR per BICR in the Asian subpopulation of CheckMate 9LA

	NIVO + IPI + chemo^a^ (*n* = 28)	Chemo^b^ (*n* = 30)
ORR, *n* (%)	16 (57)	7 (23)
Best overall response, *n* (%)		
Complete response	2 (7)	0
Partial response	14 (50)	7 (23)
Stable disease	9 (32)	16 (53)
Progressive disease	3 (11)	5 (17)
Could not be determined	0	2 (7)
Disease control rate, *n* (%)	25 (89)	23 (77)
Time to response, median (range), months	1.5 (1.2–9.5)	2.8 (1.2–5.4)
DOR, median (95% CI), months	7.0 (2.8–11.0)	4.4 (2.8–NR)

*BICR* blinded independent central review, *Chemo* chemotherapy, *CI* confidence interval, *DOR* duration of response, *IPI* ipilimumab, *n* number of patients, *NIVO* nivolumab, *NR* not reached, *ORR* objective response rate

^a^Nivolumab plus ipilimumab combined with chemotherapy (2 cycles)

^b^Chemotherapy alone (4 cycles, with optional pemetrexed maintenance for nonsquamous histology)

### Safety

TRAEs of any grade among Asian patients were reported in 28 (100%) patients treated with nivolumab plus ipilimumab combined with chemotherapy and in 29 (97%) patients treated with chemotherapy (Table 5). The most common (in ≥ 40% of patients) any-grade TRAEs included decreased appetite (46%) and constipation (43%) in patients treated with nivolumab plus ipilimumab combined with chemotherapy; and nausea (60%), constipation (57%), anemia (50%), and decreased appetite (40%) in patients treated with chemotherapy. Grade 3–4 TRAEs were reported in 16 (57%) patients treated with nivolumab plus ipilimumab combined with chemotherapy and in 18 (60%) patients treated with chemotherapy. The most common (in ≥ 10% of patients) grade 3–4 TRAEs included decreased neutrophil count (18%), maculopapular rash (11%), and decreased white blood cell count (11%) in patients treated with nivolumab plus ipilimumab combined with chemotherapy; and anemia (23%), decreased neutrophil count (17%), and decreased appetite (10%) in patients treated with chemotherapy. TRAEs of any grade leading to discontinuation of any component of the treatment regimen were reported in 21% of patients treated with nivolumab plus ipilimumab combined with chemotherapy versus 17% of patients treated with chemotherapy. Any-grade and grade 3–4 serious TRAEs were reported in 36% and 21% of patients treated with nivolumab plus ipilimumab combined with chemotherapy, and in 30% and 20% of patients treated with chemotherapy, respectively. No treatment-related deaths occurred in the nivolumab plus ipilimumab combined with chemotherapy arm; 1 grade 5 AE was reported in the chemotherapy arm as potentially related to treatment, but cause of death was recorded as unknown.

**Table 5:** Treatment-related adverse events in the Asian subpopulation of CheckMate 9LA

	NIVO + IPI + chemo^a^ (*n* = 28)		Chemo^b^ (*n* = 30)	
	Any grade	Grade 3–4	Any grade	Grade 3–4
Total patients with an event,^c^ *n* (%)	28 (100)	16 (57)	29 (97)	18 (60)
TRAEs occurring in ≥ 15% of patients in either treatment arm, *n* (%)				
Decreased appetite	13 (46)	2 (7)	12 (40)	3 (10)
Constipation	12 (43)	0	17 (57)	0
Nausea	11 (39)	0	18 (60)	0
Neutrophil count decreased	10 (36)	5 (18)	8 (27)	5 (17)
Fatigue	8 (29)	1 (4)	7 (23)	0
Malaise	8 (29)	0	8 (27)	0
Maculopapular rash	8 (29)	3 (11)	2 (7)	0
Anemia	8 (29)	1 (4)	15 (50)	7 (23)
Rash	7 (25)	2 (7)	0	0
Alopecia	6 (21)	0	8 (27)	0
White blood cell count decreased	6 (21)	3 (11)	6 (20)	2 (7)
Diarrhea	5 (18)	0	3 (10)	1 (3)
Pyrexia	5 (18)	0	2 (7)	0
Platelet count decreased	4 (14)	1 (4)	7 (23)	1 (3)
Peripheral sensory neuropathy	3 (11)	0	9 (30)	0
Hiccups	3 (11)	0	8 (27)	0
TRAEs leading to treatment discontinuation^d^, *n* (%)	6 (21)	3 (11)	5 (17)	2 (7)
Serious TRAEs^e^, *n* (%)	10 (36)	6 (21)	9 (30)	6 (20)
Treatment-related deaths^f, g^, *n*	0		0	

*Chemo* chemotherapy, *IPI* ipilimumab, *n* number of patients, *NIVO* nivolumab, *TRAE* treatment-related adverse event

^a^Nivolumab plus ipilimumab combined with chemotherapy (2 cycles)

^b^Chemotherapy alone (4 cycles, with optional pemetrexed maintenance for nonsquamous histology)

^c^Includes events reported between first dose and 30 days after last dose of study drug

^d^Includes discontinuation of any component of the regimen

^e^Serious adverse events are defined as any untoward medical occurrence that, at any dose, result in the following: death or risk of death at the time of the event; inpatient hospitalization or prolongation of existing hospitalization, with the following exceptions: a visit to the emergency room or other hospital departments < 24 h, elective surgery (planned prior to signing consent), admissions as per protocol for a planned medical/surgical procedure, routine health assessment requiring admission for baseline/trending of health status, medical/surgical admission other than to remedy ill health and planned prior to entry into the study, admission for another life circumstance that is unrelated to health status and requires no medical/surgical intervention, and admission for administration of anticancer therapy in the absence of any other serious adverse event; persistent or significant disability/incapacity; and congenital abnormalities/birth defects. Important medical events (defined as events that may not be immediately life threatening or result in death or hospitalization but, based upon appropriate medical and scientific judgement, may jeopardize the participant or require intervention to prevent other serious outcomes listed above) are also classified as serious adverse events

^f^Within 100 days of last dose

^g^One grade 5 TRAE was reported in the chemotherapy arm, but cause of death was recorded as unknown

**Fig. 3:**
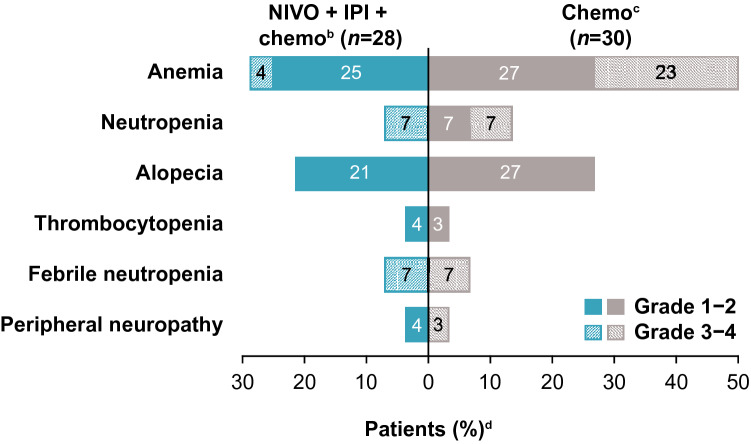
TRAEs typically associated with chemotherapy^a^ in the Asian subpopulation of CheckMate 9LA. ^a^Includes events reported between first dose and 30 days after last dose of study drug; ^b^Nivolumab plus ipilimumab combined with chemotherapy (2 cycles); ^c^Chemotherapy alone (4 cycles, with optional pemetrexed maintenance for nonsquamous histology); ^d^Data labels not shown for values < 1%. *Chemo* chemotherapy, *IPI* ipilimumab, *n* number of patients, *NIVO* nivolumab, *TRAE* treatment-related adverse event

Treatment-related select AEs, defined as AEs with potential immunologic cause, with nivolumab plus ipilimumab combined with chemotherapy were reported by organ system as follows: skin (75%), endocrine (32%), hepatic (21%), gastrointestinal (18%), pulmonary (11%), and renal (7%) (Table 6); most events were grade 1–2. Of TRAEs typically associated with chemotherapy, anemia was the most common event in both the nivolumab plus ipilimumab combined with chemotherapy and chemotherapy arms (grade 1–2, 25% and 27%; grade 3–4, 4% and 23%, respectively), followed by alopecia (grade 1–2, 21% and 27%, respectively; no grade 3–4 events were reported for either arm) (Fig. 3).

**Table 6:** Treatment-related select adverse events^a^ in the nivolumab plus ipilimumab combined with chemotherapy arm of the Asian subpopulation of CheckMate 9LA

Patients, *n* (%)	NIVO + IPI + chemo^b^ (*n* = 28)	
	Any grade	Grade 3–4
Skin	21 (75)	5 (18)
Endocrine	9 (32)	1 (4)
Hepatic	6 (21)	0
Gastrointestinal	5 (18)	0
Pulmonary	3 (11)	0
Renal	2 (7)	1 (4)

*Chemo* chemotherapy, *IPI* ipilimumab, *n* number of patients, *NIVO* nivolumab

^a^Select adverse events are those with potential immunologic etiology that require frequent monitoring/intervention; includes events reported between first dose and 30 days after last dose of study drug

^b^Nivolumab plus ipilimumab combined with chemotherapy (2 cycles)

## Discussion

Among Asian patients with advanced NSCLC in CheckMate 9LA, first-line treatment with nivolumab plus ipilimumab combined with 2 cycles of chemotherapy provided clinically meaningful OS benefit and improved PFS and ORR versus chemotherapy alone, consistent with the results observed in the all randomized patient population [median OS, 15.6 versus 10.9 months (HR 0.66; 95% CI 0.55–0.80) at a minimum follow-up of 12.7 months; median PFS, 6.7 versus 5.0 months (HR 0.68, 95% CI 0.57–0.82); ORR, 38% versus 25%] [16]. While the Asian subpopulation comprised 8% of the total all randomized patient population, baseline characteristics were generally similar between Asian patients and the all randomized population. Notably, there was a slightly higher proportion of men among Asian patients versus the all randomized population (84% versus 70%) [16], which is reflective of the higher prevalence of NSCLC reported in men versus women in Asian countries [26]. Interestingly, the survival benefit provided by nivolumab plus ipilimumab combined with chemotherapy versus chemotherapy alone was achieved despite most patients in the chemotherapy arm receiving subsequent immunotherapy. While there may be numerical differences in efficacy results between the smaller Asian subpopulation and the all randomized patient population, the benefits of nivolumab plus ipilimumab combined with chemotherapy versus chemotherapy alone in key subgroups, including those based on tumor histology or tumor PD-L1 expression, are encouraging.

Chemotherapy for NSCLC is associated with interethnic differences in survival outcomes [23]; however, data directly comparing the efficacy of immunotherapy between Asian and global populations are limited. Some studies have shown clinical benefits of first-line immunotherapy combinations in both all randomized patients and the Asian subpopulation [27–29]. In a subanalysis of the phase 3 CheckMate 227 study in Asian patients with tumor PD-L1 expression ≥ 1% or < 1%, first-line treatment with nivolumab plus ipilimumab combination therapy demonstrated durable long-term survival and clinical benefits versus chemotherapy (median OS not reached versus 22.9 months; 2-year OS rate, 53% versus 45%, respectively), consistent with OS improvements observed in the all randomized population [29]. Subanalyses of the KEYNOTE-407 study in East Asian patients and the KEYNOTE-189 study in Japanese patients also favored first-line combination immunotherapy with pembrolizumab plus chemotherapy over chemotherapy in Asian/Japanese patients with metastatic squamous or nonsquamous NSCLC, respectively, consistent with findings in the all randomized population of each study [2, 3, 27, 28]. On the other hand, in the IMpower132 trial, atezolizumab plus chemotherapy as first-line treatment for nonsquamous NSCLC showed apparent OS improvement versus chemotherapy in the Japanese subpopulation [median OS, 30.8 versus 22.2 months; HR 0.63 (95% CI 0.36–1.14)], but OS difference in the global population was not statistically significant between the treatment arms (median OS, 17.5 versus 13.6 months; *p* = 0.1546) [30, 31]. These slight differences in outcomes between the Asian and global populations may potentially be due to underlying variance in genetic or disease characteristics as well as variations in clinical practice [23].

The safety profile of nivolumab plus ipilimumab combined with chemotherapy in Asian patients was generally consistent with that for the all randomized population of CheckMate 9LA [16], with no new safety signals. Frequencies of grade 3–4 TRAEs were higher in Asian patients versus the all randomized population in both the nivolumab plus ipilimumab combined with chemotherapy (57% versus 47%) and chemotherapy alone arms (60% versus 38%), which could be attributed to the small sample size of the Asian subpopulation. However, differences between the two treatment arms were consistent between the Asian subpopulation and the all randomized population. We also observed numerically higher rates of some select TRAEs (skin, endocrine, hepatic, and pulmonary) with nivolumab plus ipilimumab combined with chemotherapy among Asian patients than in the all randomized population, which could also be attributed to the small sample size of the Asian subpopulation.

As ethnicity was not a stratification factor in CheckMate 9LA [16], unknown confounding factors may not have been accounted for in this post hoc analysis. Additionally, the small sample size for this subpopulation analysis limits the extent of the conclusions that can be drawn. However, the benefits with nivolumab plus ipilimumab combined with chemotherapy in Asian patients were consistent across efficacy outcomes, similar to those in the all randomized patient population. Studies in larger populations of Asian patients to further evaluate the efficacy and safety of nivolumab plus ipilimumab combined with chemotherapy are warranted.

In conclusion, these CheckMate 9LA results in the Asian subpopulation support the use of nivolumab plus ipilimumab combined with a limited course of chemotherapy as first-line treatment for Asian patients with advanced NSCLC.

## Data sharing

BMS policy on data sharing may be found at https://www.bms.com/researchers-and-partners/independent-research/data-sharing-request-process.html.

## Supplementary Information

Below is the link to the electronic supplementary material.

Supplementary file1 (DOCX 65 KB)
