# Supplementary material for: First-line nivolumab plus ipilimumab combined with two cycles of chemotherapy in advanced non-small cell lung cancer: a subanalysis of Asian patients in CheckMate 9LA
Source: Int J Clin Oncol. 2022 Feb 19;27(4):695–706. doi: 10.1007/s10147-022-02120-0 (PMC8956544; doi:10.1007/s10147-022-02120-0)
Supplement: Supplementary file 1 — Supplementary file1 (DOCX 65 KB) [file 10147_2022_2120_MOESM1_ESM.docx]

**Title:** First-line nivolumab plus ipilimumab combined with two cycles of chemotherapy in advanced non-small cell lung cancer: a subanalysis of Asian patients in CheckMate 9LA

**Journal:** *International Journal of Clinical Oncology*

**Authors:** Thomas John, Hiroshi Sakai, Satoshi Ikeda, Ying Cheng, Kazuo Kasahara, Yuki Sato, Yoshiro Nakahara, Masayuki Takeda, Hiroyasu Kaneda, Helong Zhang, Makoto Maemondo, Koichi Minato, Takeshi Hisada, Yuki Misumi, Miyako Satouchi, Katsuyuki Hotta, Ang Li, Abderrahim Oukessou, Shun Lu

**Corresponding author affiliation and email:** Austin Hospital, 145 Studley Road, Heidelberg, Victoria 3084, Australia; [Tom.John@petermac.org](mailto:Tom.John@petermac.org)

**Online Resource 1:** Overall survival by histology and tumor PD-L1 expression in the Asian subpopulation of CheckMate 9LA

|  | NIVO + IPI + chemo^a^ | | Chemo^b^ | | Unstratified |
| --- | --- | --- | --- | --- | --- |
|  | *n* | Median OS, mo  (95% CI) | *n* | Median OS, mo (95% CI) | Hazard ratio^c^ (95% CI)  NIVO + IPI + chemo vs Chemo |
| Histology  Squamous  Nonsquamous | 11  17 | 16.2 (13.1–NR)  NR (15.1–NR) | 9  21 | 7.3 (1.0–17.0)  NR (11.9–NR) | NA  0.38 (0.10–1.42) |
| Tumor PD-L1 expression  PD-L1 < 1%^d^  PD-L1 ≥ 1% | 12  16 | NR (NA)  16.2 (13.1–NR) | 13  17 | 17.0 (6.6–NR)  13.2 (7.1–NR) | NA  0.45 (0.17–1.21) |

^a^Nivolumab plus ipilimumab combined with chemotherapy (2 cycles)

^b^Chemotherapy alone (4 cycles, with optional pemetrexed maintenance for non-squamous histology)

^c^Hazard ratio was not reported for treatment groups with small sample sizes

^d^Includes patients with non-quantifiable tumor PD-L1 expression

*Chemo* chemotherapy*, CI* confidence interval, *IPI* ipilimumab, *mo* months, *n* number of patients, *NIVO* nivolumab, *NA* not available, *NR* not reached, *OS* overall survival, *PD-L1* programmed death ligand-1
